# Effectiveness of In‐Person Versus Online Psychoeducational Interventions With Divorced/Separated Parents: A Comparative Analysis

**DOI:** 10.1111/famp.70138

**Published:** 2026-03-09

**Authors:** Ángela Ordóñez‐Carabaño, Pilar Martínez‐Díaz, Mª. Angustias Roldán Franco, Virginia Cagigal de Gregorio

**Affiliations:** ^1^ UNINPSI Clinical Psychology Center Comillas Pontifical University Comillas Spain; ^2^ Department of Psychology Comillas Pontifical University Comillas Spain

**Keywords:** coparenting relationship, divorce, in‐person, interparental conflict, online, parent education programs

## Abstract

The dissolution of marriage frequently results in parental distress, strained coparental relationships, and adverse effects on children. Psychoeducational interventions have the potential to mitigate these effects, but there is a paucity of research exploring their comparative efficacy in in‐person versus online formats. The present study aims to assess the comparative efficacy of two intervention modalities in reducing psychological distress, mitigating interparental conflict and enhancing coparental relationships. The study evaluated 62 participants, comprising 41 in the in‐person group and 21 in the online group, over the course of 11 weekly sessions and a 6‐month follow‐up period. A battery of pre‐ and postintervention assessments was employed to evaluate a range of variables, including symptomatology, interparental conflict, coparenting, perceived support, forgiveness, and parenting style. Across formats, participants exhibited a reduction in overall symptomatology and covert conflict, accompanied by an increase in forgiveness. In the confirmatory ANOVAs, the in‐person modality showed greater improvement than the online modality for total symptomatology, depression, and forgiveness; no other interactions reached significance. The findings suggest that both modalities may offer benefits, with comparatively larger gains observed for in‐person delivery in these domains and online delivery enhancing accessibility. These results highlight the potential for tailoring intervention formats to participant needs and contexts, maximizing the reach and impact of psychoeducational programs for postdivorce adjustment.

## 1 Introduction

The prevalence of divorce and relationship breakdowns in contemporary society is a growing concern. In Europe, the divorce rate has increased twofold over the past six decades, rising from 0.8 per 1000 individuals in 1964 to 1.6 per 1000 individuals in 2024. This figure does not fully capture the extent of relationship dissolutions, as it excludes breakups involving unmarried couples (Eurostat 2024). In Spain, the 2023 divorce rate is 1.7 per 1000 inhabitants, which is slightly above the European average. Notably, more than half (50.7%) of these divorces involve families with children (INE 2024).

Research consistently shows that divorce increases the risk of emotional, behavioral, and academic difficulties in children, although outcomes vary widely. This variability is shaped not by divorce itself but by factors such as how the process unfolds and the quality of family relationships throughout (Cao et al. 2022; Emery 2011; O'Hara et al. 2019). Key family‐level influences include coparenting quality, parental distress, interparental conflict, and parenting practices (Lamela et al. 2016; O'Hara et al. 2023; van Dijk et al. 2020).

To mitigate the negative effects of divorce, various educational programs have been developed to equip parents with skills for effective parenting, emotional regulation, conflict reduction, and constructive coparenting (Herrero et al. 2020; Saini and Corrente 2024; Schramm and Becher 2020). This study evaluated the efficacy of one such program in Spain, comparing outcomes across online and in‐person formats.

### 1.1 Parental Emotional Distress and Relational Factors in the Process of Divorce

Divorce places significant emotional strain on parents, who often face elevated levels of depression, anxiety, and stress (Amato and Anthony 2014; Herrero et al. 2020), while also needing to maintain an effective coparenting relationship, which is a key factor in children's postdivorce adjustment (Lamela et al. 2016; van Dijk et al. 2020). Coparenting includes dimensions such as cooperation and conflict, with support from the ex‐partner linked to fewer child behavior problems and greater emotional security (Amato 2010; Martínez‐Pampliega, Herrero Lázaro, et al. 2021; Rejaän et al. 2022). Conversely, interparental conflict is strongly associated with poorer child mental health and is especially damaging when intense, prolonged, escalating, or when children are involved (Cao et al. 2022; Giani et al. 2025).

The effective coparenting of children following separation or divorce is frequently impeded by the presence of unresolved negative emotions, including anger, distress, and regret directed toward the former partner (Yárnoz‐Yaben 2015). The emotional distress caused by feelings of anger and resentment is frequently associated with perceived transgressions that occur during the course of the relationship. Overcoming these emotions through forgiveness is of paramount importance for fostering cooperation and reducing conflict (Kluwer et al. 2021).

Forgiveness can be conceptualized as a process of reducing negative emotions such as anger and revenge while fostering positive feelings toward the offender while simultaneously creating the psychological space for improved personal well‐being and, where relevant, more constructive relational dynamics. This process has been demonstrated to promote personal and relational well‐being (Fincham 2019; McCullough et al. 1998). In the context of divorce, the act of forgiveness toward a former spouse has been linked to a reduction in conflict and an improvement in the quality of coparenting (Yárnoz‐Yaben 2015). Nevertheless, further research is needed to elucidate the factors that influence forgiveness in this context.

The act of forgiveness following the dissolution of a marriage has been demonstrated to be associated with the formation of more harmonious coparental relationships (Bonach 2008). It moderates the relationship between the perceived causes of the dissolution of the relationship, the quality of coparenting, and conflict levels. Factors that are negatively associated with forgiveness include hostile attributions, conflict severity, narcissistic entitlement, and attachment anxiety (Kluwer et al. 2021).

### 1.2 Parenting After Divorce

Postdivorce parenting quality is strongly influenced by the emotional climate between ex‐partners and parental psychological distress. Hostile or conflicted coparenting often undermines consistency, warmth, and responsiveness in parenting (Amato 2000; O'Hara et al. 2023; van Dijk et al. 2020), whereas divorce can reduce parenting time and emotional availability (Amato 2014), increasing children's risk of maladjustment. In contrast, supportive parenting can buffer these effects and promote healthier child outcomes. As a result, many interventions include modules to strengthen parenting skills and help parents navigate childrearing amid coparental strain (Alcaniz et al. 2022; O'Hara et al. 2024; Saini and Corrente 2024; Schramm and Becher 2020; Sigal et al. 2011).

### 1.3 Divorce Education Programs for Parents

Programs for divorcing parents typically address a broad range of objectives, the most relevant of which include reducing interparental conflict, minimizing legal disputes, enhancing the quality of parenting and increasing child wellbeing (Saini and Corrente 2024; Sandler et al. 2024). Evidence suggests that such programs positively impact children's well‐being by mitigating the adverse effects of divorce and promoting a supportive environment (O'Hara et al. 2024; Turner et al. 2023). They also help parents navigate new family dynamics in a developmentally appropriate way, addressing the specific needs of both children and adults during this transition (Schramm and Calix 2011).

Program content is organized into three priority levels, with core content focusing on child‐related topics such as the impact of divorce, positive coparenting, parenting strategies, and conflict reduction (Schramm and Becher 2020). Lower‐priority content addresses adult well‐being and special circumstances, although some parents, especially those facing trauma, violence, or addiction, may need individualized support (Becher et al. 2019; Markham and Ferraro 2024). The current program targets core content areas, and both the online and in‐person formats deliver identical material, differing only in delivery method and interaction format.

Despite variations in format, duration, and content, parent education programs have been shown to be effective in improving postdivorce adjustment (Saini and Corrente 2024; Sandler et al. 2024). Programs for separated or divorced parents enhance conflict management and coparenting skills and support adjustment to separation, benefiting both vulnerable and stable families (Eira Nunes et al. 2021; Markham and Ferraro 2024; Martínez‐Pampliega, Herrero Lázaro, et al. 2021; Schramm and Calix 2011). In a recent meta‐analysis by Saini and Corrente (2024), which examined 40 parenting education programs, changes were reported across several outcome variables, including interparental conflict, coparenting knowledge, communication, parent–child relationships, child adjustment, and parental competence. Greater benefits are observed among younger parents, women, and those with lower education or socioeconomic status (Schramm and Calix 2011). Additionally, a shorter time since separation facilitates participation and improves outcomes (Tomlinson et al. 2024).

In the United States, such programs are widespread and mandatory in many states, whereas in Spain, they remain scarce and voluntary. This study contributes by implementing and evaluating a Spanish program that responds to the need for culturally specific interventions (Schramm and Becher 2020).

### 1.4 In‐Person vs. Online Interventions

Psychoeducational programs for separated or divorced parents have shown effectiveness in online formats (Mahon 2014; O'Hara et al. 2024), with no significant differences found between in‐person and hybrid formats for addressing child‐related conflict (Dealy et al. 2023). However, participation, especially in mandated settings, remains a challenge (O'Hara et al. 2024). While online delivery increases accessibility, it often results in higher dropout rates and requires adaptations in session design and follow‐up to assess long‐term impact (Guyette and Harris 2024; Mahon 2014). The pandemic has further highlighted the need for format comparisons.

Both in‐person and online therapies are effective but differ in terms of their relational dynamics. Online formats offer convenience and reduced stigma but may lack strong therapeutic alliances in in‐person settings due to limited nonverbal communication (Stubbings et al. 2013). Online group therapy can hinder trust and cohesion (Weinberg 2021), whereas in‐person formats foster connection through informal interaction and nonverbal cues (Bean et al. 2022). In high‐conflict divorce cases, online delivery may aid physical separation but poses challenges in terms of confidentiality, risk management, and attunement. Additionally, in cases of intimate partner violence (IPV), the delivery format of interventions can carry distinct implications. Online interventions have the potential to enhance safety and autonomy by allowing participants to engage from secure locations. However, they may also limit professionals' ability to assess risk and respond promptly to signs of distress. Conversely, in‐person sessions offer more opportunities for nuanced observation and immediate support. However, they can pose safety concerns if proper protective measures are not in place (Constantino et al. 2015; Emezue 2020). In this sense, format selection should be performed individually (Hertlein et al. 2015).

This study aims to evaluate the efficacy of the *Egokitzen* intervention program in reducing parental symptomatology, improving coparental relationships, enhancing parenting styles, and increasing forgiveness. Furthermore, it compares outcomes between in‐person and online delivery formats to assess their relative effectiveness and inform optimization for diverse contexts and populations.

## 2 Method

### 2.1 Design and Procedure

**FIGURE 1:**
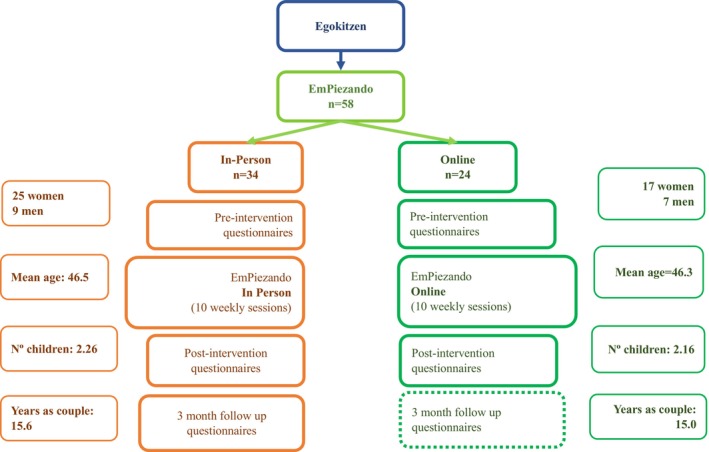
Intervention and study design.

The original intervention, designated “Egokitzen,” was developed by the research group Deusto FamilyPsych at Deusto University, Spain (Figure 1). Its efficacy has already been demonstrated in a variety of contexts with positive outcomes (Alcaniz et al. 2022; Martínez‐Pampliega, Herrero Lázaro, et al. 2021). Subsequently, a group of psychotherapists from Comillas Pontifical University and UNINPSI (Clinical Psychology Unit), trained in the original intervention, made slight adaptations and implemented the intervention called *EmPiezando*. This intervention is essentially the same as the original, retaining the objectives while modifying the name to enhance its contextual comprehension in the region of Madrid (Spain). The adapted intervention comprised 11 weekly sessions (plus a follow‐up session 6 months later), delivered in consecutive online and in‐person groups between January 2020 and December 2022 in Spain. The groups included between 7 and 10 participants. All participants completed pre, post, and 6‐month follow‐up assessments, which measured the degree of different types of conflict and the quality of the coparental relationship. At the time of data analysis for this article, follow‐up results for participants in the online intervention were not available, thus impeding the inclusion of these data in the analyses.

This research was conducted in accordance with the ethical guidelines established by the Institutional Review Board of Comillas Pontifical University, which granted formal approval (Approval No. 2019‐31 on March 19, 2019). All participants provided their informed consent before completing the questionnaires.

### 2.2 Participants

A total of 62 separated or divorced adults participated in the study. With respect to the in‐person treatment modality, 41 adults were included, 10 of whom were male and 31 of whom were female. The mean age of these participants was 46.26 years (SD = 6.00), the mean number of children they had was 2.26, and the mean time since separation was 3.98 years. In contrast, the online modality included 21 separated or divorced adults, 8 males and 13 females. The mean age was 45.37 years (SD = 4.89), the mean number of children was 2.16, and the mean time since separation was 1.01 years. The highest education levels attained by the participants were as follows: upper secondary/high school or vocational training (13.79%), short‐cycle college degree (20.69%), bachelor's degree (36.21%), and master's/doctoral degree (29.31%). In accordance with standard research practices in Spain, racial and ethnic data were not collected.

To be eligible for participation, individuals were required to meet several criteria: having at least one child regardless of the type of custody, being separated or divorced, not residing with the former partner during the intervention period, and having adequate communication and interpersonal skills. These criteria were assessed at initial contact. Participation was voluntary, and ex‐partners were placed in separate groups to reduce conflict risk, protect confidentiality, and ensure a safe space for open expression.

Exclusion criteria were established to ensure participant safety and program suitability. Individuals with severe psychopathological disorders, couples cohabiting at the same address, and those subject to restraint orders were excluded, as these circumstances could compromise the scope, integrity, or safety of the intervention. If the presence of intimate partner violence was uncertain, it was assessed during the initial contact. Those experiencing violence were excluded from participating, as the intervention itself could increase the risk of violence.

### 2.3 Intervention

*Egokitzen* is a psychoeducational and preventive program for divorced or separated parents that is delivered in groups (Martínez‐Pampliega, Sanz Vázquez et al. 2021). The overarching objective of the program is to provide parents with the knowledge and competencies required to safeguard their children from the stressors and potential conflicts associated with divorce. The program comprises 11 weekly sessions (see Supporting Information), each of which focuses on three key content areas: the intricacies of divorce itself; the dynamics of interparental conflict; and effective parenting styles, discipline, and rules. By concentrating on these elements, the *Egokitzen* program aims to provide parents with the practical tools and insights necessary to foster a supportive environment that is conducive to children's well‐being (Martínez‐Pampliega et al. 2021).

### 2.4 Measures

The participants were requested to complete a series of questionnaires designed to assess a range of variables, including symptomatology, coparental relationships, parenting style, and degree of forgiveness of the ex‐partner.

#### 2.4.1 Parents' Symptomatology

Symptomatology was measured with the Symptom Assessment‐45 Questionnaire (SA‐45; Maruish 2004), validated in Spain by Sandín et al. (2008). The SA‐45 is a 45‐item self‐report instrument derived from the SCL‐90 (Derogatis et al. 1973) and comprises nine scales, each containing five items, which assess anxiety, depression, obsessive‐compulsive symptoms, somatization, phobic anxiety, hostility, interpersonal sensitivity, paranoid ideation, and psychoticism. The instrument exhibited an overall internal consistency of 0.95. The reliability coefficients for each subscale range from 0.63 to 0.85.

#### 2.4.2 Coparental Relationship

##### 2.4.2.1 Hostility

The Brief Acrimony Scale (BACS‐8) developed by Rahimullah et al. (2020) measures interparental hostility among divorced parents. A forward‐backward translation procedure was conducted by bilingual experts for the Spanish version. The internal consistency of the 8‐item scale was high in both the original validation (*α* = 0.84) and the current Spanish sample (*α* = 0.88).

##### 2.4.2.2 Coparenting

The original version of the Multidimensional Coparenting Scale for Dissolved Relationships (MCS‐DR) developed by Ferraro et al. (2018) is a 23‐item questionnaire designed to capture the multifaceted nuances of postdivorce coparental relationships. It comprises four distinct subscales: overt conflict, support, self‐controlled covert conflict, and externally controlled covert conflict. The scale has good internal consistency, with Cronbach's alpha coefficients ranging from 0.77 to 0.92 for the four subscales (Ferraro et al. 2018). A forward‐backward translation procedure was conducted by bilingual experts for the Spanish version. The scale and its subscales have demonstrated adequate internal consistency (ranging from 0.79 to 0.94) and adequate factorial and criterion validity in the Spanish population, as evidenced by the expected correlations with conflict and the quality of the post‐divorce relationship (Ordóñez‐Carabaño et al. 2024).

##### 2.4.2.3 Support

The Ex‐Spouse Support Questionnaire (CARE Questionnaire, Yárnoz‐Yaben 2010) is an 8‐item measure developed for the Spanish population and is designed to assess the perception of the responding parent regarding the support provided by their ex‐partner in raising the children. The instrument showed excellent internal consistency (*α* = 0.91).

#### 2.4.3 Parenting Style

The Spanish version of the Short‐EMBU (Arrindell et al. 2005) was used. The short(s)‐EMBU (Swedish acronym for Egna Minnen Beträffande Uppfostran [My memories of upbringing]) consists of 23 items and contains 3 subscales (emotional warmth, overprotection and rejection), with 6, 9, and 7 items, respectively, that are scored for each parent separately. *Emotional warmth* involves evaluating physical or verbal parental displays of love, acceptance and availability; the *overprotection* subscale assesses parental attempts to control children's behavior and excessive concern for children's health and outings; and the *rejection* subscale assesses whether parents show physical or verbal hostility, aggression or indifference toward their children. The scale showed good internal consistency, with Cronbach's alpha coefficients ranging from 0.71 to 0.87 for the three subscales.

#### 2.4.4 Forgiveness

The *Forgiveness in Divorce‐Separation Questionnaire* (CPD‐S, Yárnoz‐Yaben and Comino González 2012), comprising five items, was developed with a Spanish population and included in the study to assess forgiveness in the context of divorce/separation. The instrument showed good internal consistency, with a Cronbach's alpha coefficient of 0.77. The CPD‐S exhibited a unidimensional factorial structure and substantial convergent and predictive validity, as evidenced by its correlations with divorce adjustment, life satisfaction, and perceived support from the ex‐partner. Sample items include “I have forgiven my ex‐partner” and “I can't help but blame my ex‐spouse for causing the breakup,” capturing both positive and negative aspects of forgiveness.

### 2.5 Data Analyses

All analyses were conducted in SPSS v28. To address Objective 1 (baseline associations), we used Spearman's rho (*ρ*) because most of the variables were not normally distributed according to the Shapiro–Wilk test. For Objectives 2 and 3 (pre‐post change overall and by modality), we ran a paired *t*‐test when normality was met and a Wilcoxon signed‐rank test when it was not met. Levene's test confirmed equal baseline variances. For Objective 4 (differential effectiveness by delivery mode), we estimated a mixed 2 (mode) × 2 (time) ANOVA to test the time × mode interaction. Therefore, Objectives 1 to 3 are supportive/descriptive, and Objective 4 is confirmatory.

## 3 Results

### 3.1 Preliminary Analyses

#### 3.1.1 Research Objective 1

To analyze whether coparental relationships, educational style, and forgiveness are associated with self‐reported symptomatology at the preintervention assessment:

With respect to the results, a total of 62 participants were included in the analysis. The results of the analyses indicated that the assumption of normality could not be justified for the majority of the outcome measures. Consequently, nonparametric Spearman's rho correlations were calculated to quantify the associations among all the outcome variables. We initially conducted preliminary analyses to examine the potential associations between all the measures at baseline (Table 1). The results indicated a strong negative correlation between *hostility* (BACS‐8) and perceived *support* (CARE; *ρ* = −0.893; *p* < 0.01). Additionally, *forgiveness* (CPD‐S) was inversely associated with *hostility* (BACS‐8; *ρ* = −0.482; *p* < 0.01) and *symptomatology* (SA‐45; *ρ* = −0.352; *p* < 0.01). A direct association was observed between *forgiveness* (CPD‐S) and perceived *support* (CARE; *ρ* = 0.416; *p* < 0.01). Finally, a direct association was found between the *rejection* subscale of the S‐EMBU and *symptomatology* (SA‐45; *ρ* = −0.272; *p* < 0.05).

**TABLE 1:** Spearman's rho (*ρ*) correlations among the major outcome variables at baseline (*N* = 62).

	SA‐45	BACS‐8	CARE	MCS‐DR	CPD‐S	Reject.S‐EMBU	E.Warmth S‐EMBU	Overp.S‐EMBU
SA‐45 (Sympt.)	1							
BACS‐8 (Hostility)	0.223	1						
CARE (Support)	−0.104	−0.893**	1					
MCS‐DR (Coparenting)	−0.001	0.108	−0.010	1				
CPD‐S (Forgiveness)	−0.352**	−0.482**	0.416**	0.082	1			
Rejection S‐EMBU	0.272*	0.078	−0.019	0.012	0.167	1		
Emotional warmth S‐EMBU	−0.158	0.023	−0.030	−0.153	0.068	−0.288*	1	
Overprotection/ Control S‐EMBU	0.184	−0.010	0.092	0.030	−0.129	0.439**	0.004	1

* *p* < 0.05.

** *p* < 0.01 (bilateral).

### 3.2 Total Results: *Egokitzen* Impact

#### 3.2.1 Research Objective 2

To analyze whether coparental relationships, parenting styles, and forgiveness improve and symptomatology decreases after participation in the *Egokitzen* intervention.

All measures related to symptomatology, hostility, perceived support, and forgiveness showed the expected trends following participation in the intervention (Table 2). The only measures that did not undergo a change were those related to parenting style (S‐EMBU). Additionally, several improvements reached statistical significance. Scores on the SA‐45 showed reductions across several subscales. General symptomatology decreased, *t*(61) = 3.251, *p* = 0.002, *d* = 0.38 (small). Depression decreased, *t*(61) = 3.667, *p* = 0.001, *d* = 0.50 (medium); anxiety, *t*(61) = 4.054, *p* < 0.001, *d* = 0.76 (medium); paranoid ideation, *t*(61) = 2.040, *p* = 0.049, *d* = 0.28 (small); and psychoticism, *t*(61) = 3.135, *p* = 0.003, *d* = 0.85 (large). However, only four participants scored above 0 on the Psychoticism Subscale; therefore, changes in this domain should be interpreted with great caution. Furthermore, the self‐controlled covert conflict subscale score demonstrated a notable decline from pre‐ to postintervention, *t*(61) = 2.196, *p* = 0.035, *d* = 0.29, with a small effect size. There was a slight increase in forgiveness, *t*(61) = −1.834, *p* = 0.076, *d* = 0.26 (small). These findings suggest that the *Egokitzen* intervention may help reduce some aspects of symptomatology and improve conflict levels and forgiveness when considering the full sample; however, without a control group, causal attribution cannot be established.

**TABLE 2:** Total sample analyses (*N* = 62). Means, standard deviations, Student's *t*‐tests (g.l. = 61), and effect sizes on all the normally distributed baseline and postmeasures.

Scale/Subscale	Mean (SD) Pre‐Int.	Mean (SD) Post‐Int.	*t*	*p*	Cohen's *d*
SA‐45 (Symptomatology)	0.56 (0.34)	0.42 (0.36)	3.251	0.002**	0.38***
Hostility	0.33 (0.46)	0.29 (0.45)	0.713	0.480	0.08
Somatization	0.68 (0.80)	0.56 (0.75)	0.849	0.401	0.15
Depression	1.13 (0.77)	0.80 (0.66)	3.667	0.001**	0.50*
Obsessive‐Comp	0.62 (0.48)	0.55 (0.65)	0.888	0.381	0.11
Anxiety	0.95 (0.61)	0.58 (0.48)	4.054	0.000**	0.76*
Interp. Sensitivity	0.44 (0.50)	0.41 (0.54)	0.458	0.650	0.06
Phobic Anxiety	0.15 (0.30)	0.10 (0.26)	0.801	0.428	0.18
Paranoid Ideation	0.54 (0.53)	0.40 (0.48)	2.040	0.049*	0.28***
Psychoticism	0.16 (0.24)	0.04 (0.13)	3.135	0.003**	0.85**
BACS‐8 (Hostility)	2.86 (0.69)	2.73 (0.72)	1.219	0.231	0.18
MCS‐DR (Coparenting)					
Overt conflict	2.45 (0.54)	2.44 (0.49)	0.151	0.880	0.03
Self‐controlled covert conflict	1.93 (0.64)	1.73 (0.69)	2.196	0.035*	0.29***
Externally Controlled Covert Conflict	2.04 (0.70)	1.84 (0.65)	1.492	0.145	0.30***
CARE (Support)	2.66 (0.98)	2.93 (1.01)	−1.597	0.119	0.26***
Forgiveness	13.81 (4.64)	15.27 (5.50)	−1.834	0.076***	0.26***
Rejection (S‐EMBU)	1.25 (0.29)	1.21 (0.23)	0.973	0.337	0.18
Emotional warmth (S‐EMBU)	3.72 (0.31)	3.71 (0.30)	0.165	0.870	0.03
Overprotection/control (S‐EMBU)	1.96 (0.37)	1.95 (0.31)	0.138	0.891	0.03

*Note:* Cohen's *d* < 0.20, no effect; 0.21 ≥ *d* ≤ 0.49, small effect size = +; 0.50 ≥ *d* ≤ 0.79, medium effect size = *; *d* > 0.80, large effect size = **.

* *p* ≤ 0.05.

** *p* ≤ 0.01.

^***^ *p* ≤ 0.10.

### 3.3 In‐Person and Online

#### 3.3.1 Research Objective 3

To analyze whether coparental relationships, parenting styles, and forgiveness increase and whether symptomatology decreases in both online and in‐person groups.

Tables 3 and 4 summarize the results for the in‐person and online groups. The pre‐post change was assessed via Student's *t*‐tests for paired samples, except for those cases where normality could not be assumed (checked with the Shapiro–Wilk test); for those variables, nonparametric Wilcoxon signed‐rank tests were conducted. Levene's test revealed that variances at baseline were equal in both groups (*p* > 0.05).

**TABLE 3:** Intragroup analyses: Means, standard deviations, Wilcoxon tests, and effect sizes for pre‐ and postintervention measures on all the nonnormally distributed outcome measures (in‐person vs. online).

Scale/Subscale	Group	Mean (SD) Pre (T1)	Mean (SD) Post (T2)	*Z*	*p*	*d*
SA‐45 Hostility	In‐person	1.88 (2.58)	1.29 (2.43)	−2.254	0.024*	0.25***
	Online	2.04 (2.08)	1.71 (2.02)	−0.680	0.496	0.17
SA‐45 somatization	In‐person	3.24 (3.54)	2.19 (2.60)	−2.119	0.034*	0.40***
	Online	3.09 (3.90)	3.65 (4.69)	−1.023	0.306	0.12
SA‐45 obsessive‐compulsive	In‐person	3.76 (3.22)	1.86 (2.26)	−1.902	0.057***	0.84**
	Online	3.91 (2.45)	3.82 (3.94)	−0.281	0.778	0.02
SA‐45 interp. sensitivity	In‐person	2.68 (2.52)	1.90 (2.57)	−1.799	0.072***	0.30***
	Online	2.13 (3.25)	2.24 (2.91)	−0.475	0.635	0.04
SA‐45 phobic anxiety	In‐person	0.71 (1.51)	0.33 (0.58)	−1.393	0.164	0.62*
	Online	0.61 (0.99)	0.76 (1.89)	−0.137	0.891	0.08
SA‐45 paranoid ideation	In‐person	3.29 (2.49)	1.95 (2.22)	−2.543	0.011*	0.60*
	Online	1.87 (2.55)	2.06 (2.70)	−0.359	0.719	0.07
SA‐45 psychoticism	In‐person	0.85 (1.33)	0.29 (0.78)	−1.439	0.150	0.71*
	Online	0.87 (1.18)	0.12 (0.33)	−2.585	0.010**	2.11**
BACS‐8 (hostility)	In‐person	2.66 (0.98)	2.73 (0.88)	−0.104	0.917	0.07
	Online	2.70 (0.65)	2.73 (0.46)	−1.192	0.233	0.06
CARE (support)	In‐person	2.95 (1.20)	2.88 (1.20)	−0.502	0.616	0.06
	Online	2.87 (0.86)	3.03 (0.73)	−1.891	0.059***	0.21***
S‐EMBU Rejection	In‐person	1.28 (0.23)	1.24 (0.25)	−0.729	0.466	0.18
	Online	1.28 (0.32)	1.17 (0.22)	−0.351	0.725	0.51*
S‐EMBU Emotional warmth	In‐person	3.67 (0.34)	3.71 (0.36)	−0.543	0.587	0.10
	Online	3.56 (0.60)	3.68 (0.29)	−0.080	0.937	0.41***

*Note:* Cohen's *d* < 0.20, no effect; 0.21 ≥ *d* ≤ 0.49, small effect size = +; 0.50 ≥ *d* ≤ 0.79, medium effect size = *; *d* ≥ 0.80, large effect size = **.

* *p* ≤ 0.05.

** *p* ≤ 0.01.

^***^ *p* ≤ 0.10.

**TABLE 4:** Means, standard deviations, student *t*‐tests, effect size, and interaction effects (ANOVAs) all the normally distributed measures (in‐person vs. online).

Scale/Subscale	Group	Mean (SD) pre (T1)	Mean (SD) post (T2)	Student *t*	*d.f*.	*p*	Cohen's *d*	Interaction effect (mixed design ANOVAs)		
								F (1, 19)	*p*	*η*_*p*_^*2*^
SA‐45 total	In‐person	27.88 (17.25)	15.90 (14.85)	4.721	20	0.000**	0.80**	8.735	0.006*	0.200**
	Online	24.65 (14.81)	22.06 (17.29)	5.229	20	0.000**	0.15			
SA‐45 depression	In‐person	6.62 (4.62)	3.29 (3.27)	2.887	20	0.009**	1.01**	9.394	0.004*	0.212**
	Online	5.22 (3.50)	4.76 (3.25)	0.568	20	0.576	0.14			
SA‐45 anxiety	In‐person	4.85 (3.23)	2.81 (2.32)	−0.426	20	0.674	0.88**	0.024	0.878	0.001
	Online	4.91 (2.63)	2.94 (2.46)	0.775	20	0.447	0.80**			
MCS‐DR total	In‐person	2.24 (0.40)	2.16 (0.58)	1.097	20	0.286	0.13	0.697	0.409	0.020
	Online	2.35 (0.49)	2.12 (0.39)	0.383	20	0.706	0.58*			
MCS‐DR overt conflict	In‐person	2.39 (0.50)	2.45 (0.50)	−2.519	17	0.022*	0.12	0.819	0.372	0.023
	Online	2.54 (0.60)	2.43 (0.50)	0.633	18	0.535	0.23***			
MCS‐DR support	In‐person	2.52 (0.54)	2.38 (0.76)	0.114	15	0.910	0.20	0.000	0.988	0.000
	Online	2.58 (0.43)	2.43 (0.41)	0.299	15	0.769	0.37***			
MCS‐DR self‐controlled covert conflict	In‐person	1.99 (0.62)	1.86 (0.76)	2.842	15	0.012*	0.17	0.984	0.328	0.027
	Online	1.87 (0.70)	1.58 (0.58)	2.195	15	0.044*	0.51*			
MCS‐DR externally‐controlled covert conflict	In‐person	1.89 (0.65)	1.83 (0.75)	0.981	15	0.342	0.09	1.308	0.261	0.036
	Online	2.24 (0.75)	1.87 (0.53)	1.432	15	0.173	0.68*			
CPD‐S	In‐person	13.72 (3.89)	16.72 (5.05)	2.042	15	0.059***	0.60*	5.148	0.030*	0.142**
	Online	13.93 (5.56)	13.53 (5.68)	1.809	15	0.091***	0.07			
S‐EMBU overprotection	In‐person	1.99 (0.43)	1.92 (0.30)	0.498	14	0.626	0.24***	0.829	0.369	0.025
	Online	1.93 (0.31)	1.99 (0.34)	−0.712	15	0.488	0.19			

*Note:* Cohen's *d* < 0.20, no effect; 0.21 ≥ *d* ≤ 0.49, small effect size = +; 0.50 ≥ *d* ≤ 0.79 medium effect size = *; *d* ≥ 0.80, large effect size = **. Partial eta squared *η*_*p*_^2^ ≥ 0.14 large effect = **; 0.06 ≥ *η*_*p*_^2^ ≤ 0.13, medium effect = *; 0.01 ≥ *η*_*p*_^2^ ≤ 0.05, small effect.

* *p* ≤ 0.05.

** *p* ≤ 0.01.

^***^ *p* ≤ 0.10.

With respect to the results related to symptomatology, both groups showed a significant reduction in total symptomatology (SA‐45; in‐person: *t*(20) = 4.721, *p* < 0.001, *d* = 0.80; online: *t*(20) = 5.229, *p* < 0.001, *d* = 0.15). The group that received the in‐person intervention also experienced a significant reduction in hostility (SA‐45; Wilcoxon *Z* = −2.254, *p* = 0.024, *d* = 0.25), somatization (SA‐45; *Z* = −2.119, *p* = 0.034, *d* = 0.40), obsessive‐compulsive symptomatology (SA‐45; *Z* = −1.902, *p* = 0.057, *d* = 0.84; trend), interpersonal sensitivity (SA‐45; *Z* = −1.799, *p* = 0.072, *d* = 0.30; trend), paranoid ideation (SA‐45; *Z* = −2.543, *p* = 0.011, *d* = 0.60), and depression (SA‐45; *t*(20) = 2.887, *p* = 0.009, *d* = 1.01), whereas none of these subscales changed significantly in the online group. Conversely, the online group yielded a significant reduction in psychoticism (SA‐45; *Z* = −2.585, *p* = 0.010, *d* = 2.11), which was not observed in the in‐person group (see Table 3).

With respect to the measures associated with the coparental relationship, both groups revealed a significant reduction in self‐controlled covert conflict (MCS‐DR; in‐person: *t*(15) = 2.842, *p* = 0.012, *d* = 0.17; online: *t*(15) = 2.195, *p* = 0.044, *d* = 0.51). In contrast, the online group exhibited a significant increase in perceived support from their former partner (CARE; *Z* = −1.891, *p* = 0.059, *d* = 0.21), whereas the in‐person group demonstrated no notable changes. One unexpected result is that, in the in‐person group, there was a slight but significant increase in overt conflict (MCS‐DR; *t*(17) = −2.519, *p* = 0.022, *d* = 0.12), whereas the online group showed no significant changes.

### 3.4 In‐Person vs. Online (Interaction Effect)

#### 3.4.1 Research Objective 4

To analyze whether the mean change differed significantly between the in‐person and online groups.

To assess the differential effectiveness of the *Egokitzen* intervention on the basis of the treatment modality, we conducted a mixed‐design ANOVA (2 conditions × 2 time points). The assumption of normality was verified via the Shapiro–Wilk test, and for all samples where normality could be assumed, mixed design ANOVAs were conducted with “time” (pre‐ and postassessment) as the within‐group factor and “treatment modality” (in‐person and online) as the between‐group factor. This was done to test whether one group showed greater change from pre‐ to postassessment than the other (time*treatment modality interaction).

The results revealed significant improvements in the in‐person group compared with the online group (interaction effect; Table 4). First, while both groups showed a notable reduction in their overall symptomatology scores (SA‐45) between the pre‐ and postassessment, this reduction was significantly greater in the group that received the in‐person treatment (*F*(1.19) = 8.735, *p* = 0.006, *ηp* ^*2*^ = 0.200). A similar pattern was observed for scores on the depression subscale of the SA‐45, with a significantly greater reduction observed in the in‐person group (*F*(1.19) = 9.394, *p* = 0.004, *ηp* ^*2*^ = 0.212). Finally, both groups significantly increased their levels of forgiveness after the intervention (CPD‐S). However, this increase was significantly more pronounced in the in‐person group (*F*(1.19) = 5.148, *p* = 0.030, *ηp* ^*2*^ = 0.142). All of them have large effect sizes.

## 4 Discussion

The present study evaluated the effectiveness of *Egokitzen*, a psychoeducational intervention designed for separated/divorced parents. The aim of this study was to examine changes in psychological symptomatology, coparenting dynamics, parenting style, perceived support, and forgiveness. Additionally, a comparison of outcomes across two delivery modalities, online and in‐person, was conducted.

### 4.1 *Egokitzen* Impact

The participants showed reductions in general psychological symptoms, especially depression, anxiety, paranoid ideation, and psychoticism, which is consistent with evidence that postdivorce education supports emotional adjustment (Amato and Anthony 2014; Sandler et al. 2024). Although the program primarily targets parenting and interparental conflict (Lamela et al. 2016), these mental health improvements likely reflect indirect effects driven by reduced relational tension and better emotional regulation (Sandler et al. 2020).

Given *Egokitzen*'s focus on postdivorce stress and conflict, reductions in depression, hostility, and anxiety were expected, whereas changes in obsessive‐compulsive symptoms or psychoticism were less predictable and should be interpreted cautiously, especially since few participants scored in clinical ranges. These effects may reflect external factors such as concurrent therapy. No significant changes in parenting style were found, likely due to the indirect nature and delayed emergence of such effects, which is consistent with prior studies (Becher et al. 2019; Sigal et al. 2011).

Overall, the results suggest that *Egokitzen* may support parents' psychological adjustment, communication, and conflict resolution, thereby promoting positive coparenting and a stable postdivorced family environment, which could contribute to improved mental health outcomes for both parents and children (Sigal et al. 2011). Specifically, perceived support from the ex‐partner increased exclusively in the online group. One potential explanation for this finding is that the structured and emotionally distanced nature of online sessions enabled participants to process interactions with their ex‐partners with reduced emotional reactivity. This may have fostered more constructive interpretations and an increased perception of support (Emezue 2020). Meanwhile, the in‐person group exhibited a slight increase in externally controlled covert conflict. This may be indicative of an elevated awareness concerning dysfunctional dynamics that have come to the fore during in‐person group interactions. During these interactions, participants were exposed to the experiences of their peers and to reflections led by the facilitator, which rendered insubstantial forms of conflict more perceptible (Sullivan et al. 2023). These format‐specific effects underscore the necessity of tailoring interventions to individual needs and contexts.

The study suggests that the online *Egokitzen* program, facilitated by a professional, may be an effective means of improving support from ex‐partners and coparenting skills, particularly in the context of self‐controlled covert conflicts. These outcomes are consistent with those observed in other programs designed to enhance coparenting after separation or divorce (Ferraro et al. 2016; Mahon 2014; Sandler et al. 2024; Turner et al. 2023).

### 4.2 In‐Person Versus Online

Additionally, ANOVA revealed that only general symptomatology, depression, and forgiveness had significant interaction effects, favoring the in‐person format. This may be because in‐person settings better support emotional connections and therapeutic alliances, whereas online formats, although more accessible, may lack these relational benefits (Stubbings et al. 2013; Weinberg 2021).

We also observed prepost reductions in self‐controlled covert conflict (MCS‐DR) in both formats, whereas externally controlled covert conflict did not change significantly. Covert conflict is understood as a subtle but harmful dynamic that undermines the authority of one parent, places children in the middle, and is linked to depression and antisocial behavior (Bradford et al. 2008; Kelly 2012). However, in the confirmatory ANOVAs, interactions for the covert conflict subscales were not significant, indicating no differential improvement between formats.

The in‐person *Egokitzen* program was associated with notable pre‐post improvements in symptomatology, including a reduction in hostility, somatization, obsessive‐compulsive symptoms, interpersonal sensitivity, paranoid ideation, depression, and psychoticism. However, the observed effects may be regarded as a result of the initial low scores or the phenomenon of regression toward the mean (Khan and Olivier 2025).

While the online group also demonstrated improvements in general symptomatology, these improvements were less pronounced than those in the in‐person group, with the exception of psychoticism (Turner et al. 2023). Both groups demonstrated an increase in forgiveness levels, with the in‐person group exhibiting greater growth. This finding is consistent with in‐person programs potentially being more efficacious for the alleviation of distress in nonclinical populations.

The Egokitzen program is designed to enhance communication and coparenting abilities rather than to directly treat psychological symptoms or address the process of forgiveness. Accordingly, the most consistent improvements were observed in these targeted areas. In addition, in‐person participants may have benefited from a stronger therapeutic alliance, which can enhance overall engagement and facilitate deeper exploration of personal issues across intervention domains (Stubbings et al. 2013).

## 5 Conclusions

Participants in the *Egokitzen* intervention showed positive changes, with a reduction observed in all measures of symptomatology, hostility, and interparental conflict. However, increases in perceived support from former partners were exclusively observed in the online group. Conversely, a marginal rise in externally controlled covert conflict was observed in the in‐person format, potentially explained by increased awareness of interparental dynamics. The findings revealed that both groups exhibited an increase in forgiveness, while no alterations were detected in perceived parenting style.

The in‐person format showed greater reductions in general symptomatology and specific domains such as hostility, somatization, paranoid ideation, psychoticism, depression, and both overt and self‐controlled covert conflict. The online format also improved general symptomatology, psychoticism, and covert conflict, with the added benefit of increased perceived support from ex‐partners. However, improvements in symptomatology, depression, and forgiveness were significantly greater in the in‐person group.

### 5.1 Limitations and Future Research

Importantly, this study is not without limitations, which should be addressed in future research. The absence of a control group and randomized samples limits the ability to draw definitive causal conclusions. Changes in symptoms or coparenting could also be interpreted as associations that may reflect the passage of time, the presence of concurrent services (e.g., individual therapy), or evolving parenting arrangements. Including a control group and using randomized samples in future studies could increase the validity of the findings. Second, this study lacked follow‐up results, which are crucial for determining the long‐term effects of the interventions. Future research should therefore incorporate follow‐up assessments to evaluate the sustainability of these changes. Third, self‐selection bias may have influenced the results, as participants may be more motivated than the general population. Parents in high‐conflict or court‐mandated settings may face participation barriers, so future research should examine outcomes in those contexts.

Finally, the relatively limited sample size of this study may restrict the generalizability of the results. It would be beneficial for future studies to include more extensive and diverse samples, thus enhancing the applicability and generalizability of the findings to broader populations.

## Conflicts of Interest

The authors declare no conflicts of interest.

## Supporting information


**Data S1:** Supporting Information.

## Data Availability

The data that support the findings of this study are available from the corresponding author upon reasonable request.
