# Supplementary material for: Effectiveness of In‐Person Versus Online Psychoeducational Interventions With Divorced/Separated Parents: A Comparative Analysis
Source: Fam Process. 2026 Mar 9;65(1):e70138. doi: 10.1111/famp.70138 (PMC12971250; doi:10.1111/famp.70138)
Supplement: Supplementary file 1 — Data S1: Supporting Information. [file FAMP-65-0-s001.docx]

**Supplemental Material**

**Table 1**

*Egokitzen Intervention Structure*

|  | **Session** | **Topic** | **Aim** |
| --- | --- | --- | --- |
| Presentation | 0 | Presentation | Getting to know the group participants and how the program works |
|  | 1 | Frequency, myths, and reactions to divorce | Review and learn some facts, statistics, and common myths about divorce, as well as common reactions of children |
| Conflict | 2 | In war zone | Promote empathy and work through interparental conflicts |
|  | 3 | Identify anger triggers | Work on managing and coping with conflict situations |
|  | 4 | Reducing anger | Reinforce skills from session 3 and practice relaxation exercises |
|  | 5 | Conflict resolution | Identify, practice, and experience communication and relaxation skills for conflict resolution |
| Parenting | 6 | General Parenting Guidelines | Understanding parenting styles I |
|  | 7 | Myths and facts about bad behavior | Understanding parenting styles II |
|  | 8 | Active listening | Promote empathy for children and communication strategies to improve parent‒child relationships |
|  | 9 | Keys to positive and effective discipline | Learn techniques and strategies for positive discipline on a daily basis |
|  | 10 | Maintain the changes learned and develop family identity | Maintain the changes learned and evaluate the group process |
